# Effects of Brown Rice and White Rice on Expression of Xenobiotic Metabolism Genes in Type 2 Diabetic Rats

**DOI:** 10.3390/ijms13078597

**Published:** 2012-07-10

**Authors:** Mustapha Umar Imam, Maznah Ismail

**Affiliations:** 1 Laboratory of Molecular Biomedicine, Institute of Bioscience, Universiti Putra Malaysia, Serdang 43400, Selangor, Malaysia; E-Mail: mustyimam@gmail.com; 2 Department of Nutrition and Dietetics, Faculty of Medicine and Health Sciences, Universiti Putra Malaysia, Serdang 43400, Selangor, Malaysia

**Keywords:** rice, drug biotransformation, gene expression, cytochrome P450

## Abstract

Xenobiotics constantly influence biological systems through several means of interaction. These interactions are disturbed in type 2 diabetes, with implications for disease outcome. We aimed to study the implications of such disturbances on type 2 diabetes and rice consumption, the results of which could affect management of the disease in developing countries. In a type 2 diabetic rat model induced through a combination of high fat diet and low dose streptozotocin injection, up-regulation of xenobiotic metabolism genes in the diabetic untreated group was observed. Xenobiotic metabolism genes were upregulated more in the white rice (WR) group than the diabetic untreated group while the brown rice (BR) group showed significantly lower expression values, though not as effective as metformin, which gave values closer to the normal non-diabetic group. The fold changes in expression in the WR group compared to the BR group for Cyp2D4, Cyp3A1, Cyp4A1, Cyp2B1, Cyp2E1, Cyp2C11, UGT2B1, ALDH1A1 and Cyp2C6 were 2.6, 2, 1.5, 4, 2.8, 1.5, 1.8, 3 and 5, respectively. Our results suggest that WR may upregulate these genes in type 2 diabetes more than BR, potentially causing faster drug metabolism, less drug efficacy and more toxicity. These results may have profound implications for rice eating populations, constituting half the world’s population.

## 1. Introduction

The ubiquitous nature of xenobiotics makes it impossible for most biological systems to evade interacting with them. In humans, contact with xenobiotics in the form of ingested food, medication, occupational exposure and many more keeps the xenobiotic metabolising enzymes constantly working to rid the body of unwanted forms or excesses of these chemical substances through biotransformation reactions [1]. In type 2 diabetes, medications are used to reverse symptomatology and improve quality of life. Already the disease is assuming alarming proportions with a projected increase in prevalence mostly in low- and middle-income countries [2]. Diabetics are likely to be on several drugs at various points of their lives, for management of hyperglycemia, hypertension, infections, hyperlipedemia and many more [3], and the cytochrome P450 (Cyp450) system helps to biotransform and remove these drugs and other xenobiotics from the body [1]. However, xenobiotic metabolising enzymes are generally limited and have wide substrate affinity, making drug-drug interactions possible [4] especially in metabolically deranged states. Xenobiotic metabolising enzymes have been reported to be dysregulated much like other metabolic perturbations in type 2 diabetes [5], resulting in disturbances of drug metabolism [6]. Specifically, Cyp2E1, Cyp4A2, Cyp2A1, Cyp2C6, Cyp2C7, Cyp3A2, Cyp4A3 and others were reported to be induced in hepatic microsomes of diabetic mice and rats [7,8]. Wang *et al*. (2003) also reported induction of Cyp2E1 in obese diabetic humans [9]. For a diabetic, therefore, any xenobiotic that would further deteriorate these perturbations could affect drug management and worsen the disease.

Over half the world’s population depends on white rice (WR) as their staple diet [10], and despite risks associated with high glycemic index foods, even diabetics in parts of Asia and Africa may have to consume it. WR has been linked to development of type 2 diabetes mellitus [11], and its high glycemic index [12] may worsen the disease if present. Brown rice (BR) has been shown to possess bioactive compounds with anti-diabetic properties [13]. Already, reports of improved glycemic control in diabetics and even reduced risk of developing diabetes in healthy people [13–15] are reasons why WR consumption should be reduced and replaced by BR. In fact, it is likely that switching to BR from WR may help reduce the burden of type 2 diabetes, and this is a challenge for rice eating populations. We made an interesting observation upon screening the effects of WR and BR consumption on expression of xenobiotic metabolism genes (Cyp2D4, Cyp3A1, Cyp4A1, Cyp2B1, Cyp2E1, Cyp2C11, UGT2B1, ALDH1A1 and Cyp2C6), the results of which provide insight into their potential effects on xenobiotic metabolism in type 2 diabetic rats. This could likely increase corresponding enzyme concentrations leading to faster metabolism, potential toxicities and less pharmaceutical efficacy.

## 2. Results and Discussion

### 2.1. Blood Glucose and Weight

WR has high insulin and glycemic indices, and may increase the risk of cardiovascular diseases, and other metabolic disorders including type 2 diabetes [16]. In fact, Sun *et al*. (2010) reported that the risk of developing type 2 diabetes was increased in people who consume WR, and those who replaced at least 50 g/day with BR had a reduced risk of developing the disease by 16% [14]. Other studies support these findings [13,15], and Hu *et al*. (2012) made a similar conclusion from a meta-analysis [11]. The benefits derived from BR are believed to be a consequence of the synergistic effect of its bioactives. Oryzanol, ferrulates, gamma-amino butyric acid, dietary fibre and other bioactives present in BR have individually been linked to many functional effects [17–20], suggesting that functional properties of BR result from cummulative effect of these bioactives in line with the concept of food synergy.

**Figure 1:**
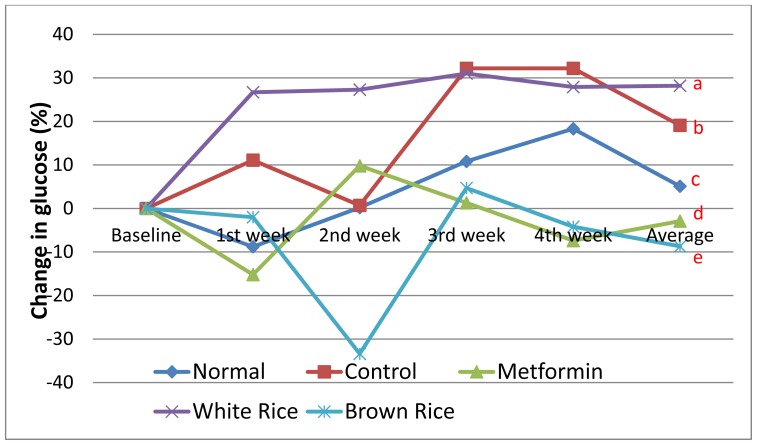
Changes in fasting blood glucose over 4 weeks of intervention; figure shows the effect of brown rice (BR) on fasting blood glucose in type 2 diabetic rats over 4 weeks of dietary intervention, as compared to white rice (WR) and metformin (n = 5). Different letters at the end of each line indicate significant difference (*p* < 0.05). Control (diabetic untreated) and normal (non-diabetic) groups received high fat diet (HFD) and normal rat chow, respectively, while the metformin group received HFD + 300 mg/kg metformin. WR and BR groups received HFD in which 50% of the normal rat chow used to formulate the pellets was substituted with 50% of the respective rice varieties.

Figure 1 shows changes in blood glucose over a 4-week intervention period. After 28 days of feeding, blood glucose had increased by an average of 19% in diabetic untreated group and by 28% in WR group, despite similarities in food consumption as shown in Table 1. This suggests that the high glycemic index of WR contributed to higher blood glucose in the latter group. Hu, Dam & Liu (2001) had reported that foods with higher glycemic and insulin indices were risk factors for type 2 diabetes and could also worsen the disease [21]. Ludwig *et al*. (1999) had also reported how consumption of foods with high glycemic index lead to problems like obesity [22], while in a meta-analysis Brand-Miller *et al*. (2003) reported that foods with low glycemic index could be useful in the management of type 2 diabetes with similar benefits as oral hypoglycemic agents [23]. The normal non-diabetic group had normal blood glucose all through the period of intervention while metformin, which is a drug used to manage type 2 diabetes mellitus [24] reduced blood glucose by 3%. BR had a greater effect than metformin and reduced blood glucose by 9% by the end of the 4 week period.

**Figure 2:**
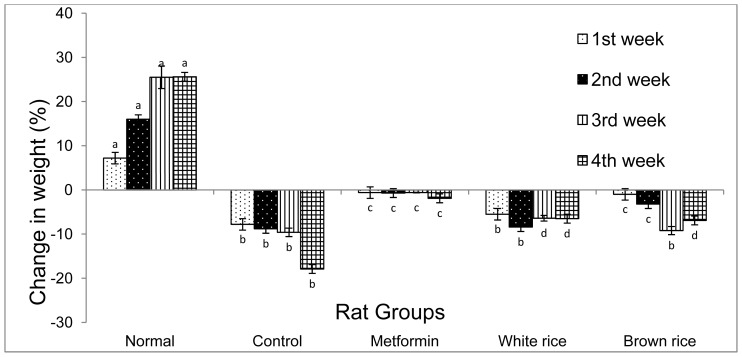
Change in weight of rats over 4 weeks of intervention; the figure shows the effect of brown rice (BR) on weight in type 2 diabetic rats over 4 weeks of dietary intervention, as compared to white rice (WR) and metformin (*n* = 5). Bars and error bars represent mean and standard deviation. Similar letters on bars representing the same week for different rat groups denote no statistical significance. Groupings are the same as in Figure 1.

Over the same period, diabetic untreated rats lost over 18% of their weight as seen in Figure 2, probably due to ongoing gluconeogenesis that used up the fat and protein deposits to make more glucose. Rats in the metformin and WR group lost approximately 9% of their weight, suggesting that metformin reduced gluconeogenesis leading to sparing of fat and protein deposits, while WR likely caused increased deposition of excess carbohydrates as fats, contrary to expectations, perhaps due to its high glycemic index. The process of gluconeogenesis is accelerated in type 2 diabetes because fat and protein are preferentially broken down to produce more glucose and energy to protect body cells from the state of starvation despite excess glucose. Under these circumstances, the WR group was expected to lose weight significantly more than the diabetic untreated group considering its glycemic control but its moderate weight loss could have been due to deposition of excess glucose as fat in adipose tissues. Though unexpected, at a certain threshold, excess blood glucose will be removed from the body, which includes storing fat in adipose tissue. This gave an impression of minimal weight loss in the WR group compared to the diabetic untreated group, contrary to expectations, because weight loss was likely compensated for by redeposition of more fat after mobilization of fats from adipose tissues to be used for energy. The BR group maintained blood glucose near normal with only a 3% weight loss over the 4 week period of intervention suggesting that BR suppressed gluconeogenesis even more than metformin, and prevented fat and protein mobilisation from stores.

### 2.2. Effect of Brown Rice on Expression of Hepatic Xenobiotic Metabolism Genes

There was an upregulation of the xenobiotic metabolism genes in the diabetic untreated group over normal non-diabetic group to varying degrees. Generally, most genes were upregulated more in the WR group compared to the diabetic untreated group except for ALDH1A1, Cyp2C6 and Cyp4A1. Interestingly, the BR group did not show as much induction of the xenobiotic metabolism genes as the WR group. Higher expression of these genes in the BR group than in the metformin group likely suggests the multiple bioactives in BR acted synergistically to upregulate the genes more. Upregulation of these genes will obviously have consequences on xenobiotic metabolism as reported previously [7], though findings by Shimojo *et al*. (1993) showed that Cyp2C11 was downregulated [8], contrary to our finding. ALDH1A1 and Cyp2E1 encode proteins that are involved in alcohol metabolism [25] and Cyp2D4, which is a rat analogue of the human CYP2D6 is involved in a wide range of xenobiotic biotransformations and is sometimes used to determine drug response in humans based on their rate of drug metabolism dependent on the particular CYPD6 phenotype [26]. Cyp2E1, in addition, is also involved in metabolism of other xenobiotics. CYP3A1 is the most extensively expressed of the CypP450 system in the liver and is said to be involved in the metabolism of a large number of drugs in common use including acetaminophen, codeine, diazepam and some steroids and carcinogens [27,28]. Over 100 therapeutic drugs are metabolized by Cyp2C11, an analogue of CYP2C9, including drugs with very narrow therapeutic index like phenytoin and other routinely prescribed drugs such as losartan, some nonsteroidal anti-inflammatory drugs and even oral hypoglycemic agents used in managing type 2 diabetes mellitus [27,29–31]. Ugt2B1 functions in the glucuronidation of endogenous metabolites, xenobiotics and drugs [32]. Aside from xenobiotic metabolism, the enzymes which these genes encode also take part in synthesis of cholesterol, steroids and other lipids [27]. Fold changes in expression of the xenobiotic metabolism genes for WR and BR groups in relation to normal non-diabetic, diabetic untreated and metformin groups are presented on Table 2.

Generally speaking, the Cyp P450 system takes part in phase 1 biotransformation reactions while UDP-glucuronosyltransferase in phase 2 [4]. In the context of drug metabolism, our results suggest that WR could hasten drug metabolism by upregulating gene expression, thereby leading to loss of drug efficacy, drug toxicity or resistance. Drugs that are active in their primary forms which need to be metabolized to become inactive will understandably lose their activity when they are metabolized faster while those that become active only after being metabolized will be produced in excess in case of an upregulation. This will mean more active metabolite at sites of drug action and an increased likelihood of toxicity. Antibiotic medication is common in type 2 diabetes mellitus due to a compromised immunity and when micro-organisms are exposed to multiple subtherapeutic doses of a particular drug, probably due to faster rate of metabolism leading to faster drug clearance, they could develop resistance to the drugs.

Drugs are taken for many conditions in type 2 diabetes and, often times, in low- and middle-income countries WR is consumed as a staple food even by diabetics. However, diabetes will lead to perturbations in drug metabolism as reported earlier [5] and corroborated by our findings. Consumption of WR, therefore, could further derange drug metabolism due to induction of xenobiotic metabolism genes, unlike BR. These findings may have implications for low- and middle-income countries where diabetes is projected to rise over the next few decades, and WR is the staple food for the majority of people.

## 3. Experimental Section

### 3.1. Chemicals

Tris-EDTA (TE) buffer solution, Sodium Chloride and Streptozotocin (STZ) used in this study were purchased from Sigma-Aldrich (St. Louis, MO, USA). Hydrogen peroxide (H_2_O_2_) was from Bendosen Laboratory Chemicals (Selangor, Malaysia), Sodium hypochlorite from Dexchem Industries Sdn. Bhd, (Penang, Malaysia) and Nespray fortified milk powder from Nestle Manufacturing (Malaysia). Fine sugar and starch powder were purchased from R & S Marketing Sdn. Bhd. (Malaysia), Mazola oil from Unilever (Malaysia) and Standard rat chow was obtained from Specialty feeds (TN, USA). Glucose strips used on accu-chek glucometer were purchased from Roche Diagnostics (Indianapolis, IN, USA)and metformin was purchased from Pfizer (USA). RCL2 Solution was purchased from Alphelys (Toulouse, France) while GenomeLab™ GeXP Start Kit was from Beckman Coulter Inc (USA).

### 3.2. Rice Grain

WR and BR from Malaysian rice variety (MR220) used in this study were supplied by PadiBeras Nasional (BERNAS) (Selangor, Malaysia). WR and BR grains were ground using a stainless steel grinder (Waring Commercial, Torrington, CT, USA) to a fine powder and used to make rat pellets.

### 3.3. Animal Handling, Feeding and Induction of Diabetes

High fat diet (HFD) was formulated according to Levin *et al*. (1989) [33] containing 47.7% total carbohydrate, 16.1% protein, 31.1% fat, 2.5% fiber and 5.1% mineral and vitamin mix, as shown in Table 1. Every kg of HFD was prepared from a mixture of 50% normal rat pellet, 24% of corn oil (Mazola brand), 20% of full-cream milk powder (Nespray brand from Nestlé), 6% sugar and 50 g of starch (to cement the pellet together). The HFD was dried in an incubator at 60 °C for 24 h, cut into small equal sized pieces and fed to the rats to induce obesity. WR and BR pellets (50% WR-HFD and 50% BR-HFD) were made by substituting 50% of normal pellet in HFD with powders of respective grains.

Twenty five male Sprague–Dawley rats (8–10 weeks old, 150–200 g) were used in this study. They were purchased from the Faculty of Veterinary Medicine, Universiti Putra Malaysia (Serdang, Selangor, Malaysia). The whole animal study was done in the animal house, Faculty of Medicine and Health Science, Universiti Putra Malaysia. Rats were individually housed in plastic cages in a controlled air conditioned room (25–30 °C) with exposure to 12/12-h light/dark cycle. Study was carried out according to the guidelines for the use of animals and was approved by the Animal Care and Use Committee (ACUC) of the Faculty of Medicine and Health Sciences, Universiti Putra Malaysia.

Rats were allowed to acclimatise to their new environment for two weeks and fed with normal rat pellets. After acclimatisation, 5 rats were continuously fed with normal rat chow while the rest were fed with HFD for six weeks to induce obesity. The obese rats were injected with low dose STZ (35 mg/kg b.w, i.p) to finally induce diabetes while the normal non-diabetic rats were injected with 5mmol/L of sodium citrate buffer (pH 4.5) [34]. Diabetic rats (fasting glucose of >250 mg/kg after 2 days of STZ injection) were divided randomly into 4 groups of 5 rats each; diabetic untreated group received HFD diet (without treatment), metformin group received HFD and 300 mg/kg/day of metformin, WR group received 50% WR-HFD diet and BR group fed with 50% BR-HFD diet. Dietary intervention lasted for 28 days, and weight was recorded at baseline and at weekly intervals thereafter.

### 3.4. Glucose Analysis

Blood glucose was determined using fasting blood samples from rat tail veins at baseline after induction of type 2 diabetes and at weekly intervals, using an Accu-Chek glucometer (Roche).

### 3.5. RNA Isolation

Rats were sacrificed after 4 weeks of intervention and their livers preserved in RCL2^®^ Solution (ALPHELYS, France) within 5–10 min of death. RNA was isolated from frozen liver samples using the GF-TR-100 RNA Isolation Kit (Vivantis, Malaysia) according to the manufacturer’s instructions. The extracted RNA was only used when it had absorbance readings for A260/230 and A260/280 falling between 1.8 and 2.0, with distinct 28 s and 18 s bands on gel electrophoresis.

### 3.6. cDNA Synthesis and PCR Amplification

RNA (50 ng) samples were reverse-transcribed using multiplex universal reverse primers (Table 3) from rat multitox plex kit purchased from Beckman Coulter Inc (USA). The reverse transcription and subsequent PCR reactions were performed according to GenomeLab™ GeXP Start Kit from Beckman Coulter protocol.

### 3.7. GeXP Multiplex Data Analysis

Each PCR product (l μL) was added to 38.5 μL sample loading solution along with 0.5 μL of DNA size standard 400 (GenomeLab GeXP Start Kit; Beckman Coulter, Inc) and analysed on a GeXP genetic analysis system (S.Kraemer Boulevard, USA).

### 3.8. Fragment Analysis and Gene Expression Signature Analysis

The data was initially analyzed using the Fragment Analysis module of the GeXP system software, then imported into the analysis module of eXpress Profiler software. Peptidylprolyl isomerase A (Ppia) was used for normalization because of consistency. Fold change in gene expression was calculated by dividing the expression values of WR or BR by those of diabetic control, normal and metformin.

## 4. Conclusions

We have been able to demonstrate that some xenobiotic metabolism genes are upregulated in type 2 diabetes mellitus and WR upregulates them even more. BR downregulates the expression of these genes when compared to WR but not as effectively as metformin. These findings could have implications for diabetics in rice eating populations especially on multiple medications, and are worth studying further.

## Figures and Tables

**Table 1:** Baseline parameters for rat groups just after induction of diabetes.

Rat groups	Body weight (g) #,*	Blood glucose (mmol/L)*	Dietary composition					Food consumption (kcal/100 g body weight/day)*

			Calories (Kcal/100 g pellet)	Total carbohydrate (%)	Protein (%)	Fat (%)	Vitamins and minerals mix (%)	
Normal non-diabetic	278 ± 16 ^a^	4.6 ± 0.5 ^a^	335	59.4	20.0	4.8	5.1	30.5 ± 3.7 ^a^
Diabetic untreated	337 ± 23 ^b^	14.9 ± 2.2 ^b^	548	47.7	16.1	31.1	5.1	34.0 ± 6.0 ^a^
Metformin	356 ± 28 ^b^	14.7 ± 4.1 ^b^	548	47.7	16.1	31.1	5.1	30.7 ± 6.0 ^a^
White rice	329 ± 16 ^b^	19.1 ± 2 ^b^	554	47.7	16.1	31.1	5.1	33.2 ± 8.3 ^a^
Brown rice	364 ± 18 ^b^	18.4 ± 2.8 ^b^	554	47.7	16.1	31.1	5.1	30.5 ± 6.7 ^a^

# Weight taken after induction of diabetes;

* Values represent mean ± SD. Values with the same letter in any given row are not statistically different (*p* > 0.05).

**Table 2:** Fold changes in expression of xenobiotic metabolism genes for white rice (WR) and brown rice (BR) groups in relation to normal, diabetic and metformin groups (*n* = 5).

Gene (Accession number)	*Fold change in relation to rat groups		

	Normal non-diabetic	Diabetic untreated	Metformin
Cyp2D4	WR 3.6	WR 1.6	WR 5.2
	BR 1.4	BR 0.6	BR 2
Cyp3A1	WR 6	WR 3.5	WR 5.5
	BR 3	BR 1.7	BR 2.6
Cyp4A1	WR 15	WR 1	WR 4.5
	BR 10	BR 0.7	BR 3
Cyp2B1	WR 20	WR 6.5	WR 13.7
	BR 4.7	BR 1.6	BR 3.3
Cyp2E1	WR 10	WR 5	WR 5.7
	BR 3.7	BR 1.7	BR 2
Cyp2C11	WR 1.8	WR 1.2	WR 2.7
	BR 1.2	BR 0.8	BR 1.8
UGT2B1	WR 4.2	WR 4.2	WR 4.2
	BR 2.4	BR 2.4	BR 2.4
ALDH1A1	WR 15	WR 0.8	WR 3.8
	BR 5	BR 0.3	BR 1.3
Cyp2C6	WR 17	WR 1	WR 5
	BR 3.7	BR 0.2	BR 1

* Fold changes for brown rice (BR) and white rice (WR) groups were calculated by dividing the expression value for BR or WR group by expression value for the group represented by each column (normal non-diabetic, diabetic untreated or metformin).

**Table 3:** Xenobiotic metabolism genes from Beckman Coulter’s (USA) rat multitox plex kit.

Accession number	Genes
NM_017101	peptidylprolyl isomerase A (Ppia)*
NM_175837	Cyp4a1
J00719	Cyp2b1
L24207	Cyp3A1
NM_031543	Cyp2e1
J02657	Cyp2c11
NM_138515	Cyp2d4
NM_173295	Ugt2B1
AF001898	Aldh1A1
M13711	Cyp2C6
NM_031144	actin, beta (Actb)*
NM_017008	glyceraldehyde-3-phosphate dehydrogenase (Gapd)*

* Housekeeping genes.

## References

[1] Xu C., Li C.Y.T., Kong A.N.T. (2005). Induction of phase I, II and III drug metabolism/transport by xenobiotics. Arch. Pharm. Res.

[2] World Health Organization Diabetes fact sheet.

[3] Stumvoll M., Goldstein B.J., van Haeften T.W. (2005). Type 2 diabetes: Principles of pathogenesis and therapy. Lancet.

[4] Lewis D.F. (2004). 57 varieties: The human cytochromes P450. Pharmacogenomics.

[5] Barnett C.R., Flatt P.R., Ioannides C. (1994). Modulation of the rat hepatic cytochrome P450 composition by long-term streptozotocin-induced insulin-dependent diabetes. J. Biochem. Toxicol.

[6] Nobuo S. (1994). Cytochrome P450 changes in rats with streptozocin-induced diabetes. Int. J. Biochem.

[7] Sakuma T., Honma R., Maguchi S., Tamaki H., Nemoto N. (2001). Different expression of hepatic and renal cytochrome P450s between the streptozotocininduced diabetic mouse and rat. Xenobiotica.

[8] Shimojo N., Ishizaki T., Imaoka S., Funae Y., Fuji S., Okuda K. (1993). Changes in amounts of cytochrome P450 isozymes and levels of catalytic activities in hepatic and renal microsomes of rats with streptozocin-induced diabetes. Biochem. Pharmacol.

[9] Wang Z., Hall S.D., Maya J.F., Li L., Asghar A., Gorski J.C. (2003). Diabetes mellitus increases the *in vivo* activity of cytochrome P450 2E1 in humans. Br. J. Clin. Pharmacol.

[10] Khush G. (2003). Productivity improvements in rice. Nutr. Rev.

[11] Hu E.A., Pan A., Malik V., Sun Q (2012). White rice consumption and risk of type 2 diabetes: Meta-analysis and systematic review. Br. Med. J.

[12] Miller J.B., Pang E., Bramall L. (1992). Rice: A high or low glycemic index food?. Am. J. Clin. Nutr.

[13] Panlasigui L.N., Thompson L.U. (2006). Blood glucose lowering effects of brown rice in normal and diabetic subjects. Int. J. Food Sci. Nutr.

[14] Sun Q., Donna S., Rob M.V.D., Michelle D.H., Vasanti S.M., Walter C.W., Frank B.H. (2010). White rice, brown rice, and risk of type 2 diabetes in US men and women. Arch. Intern. Med.

[15] Zhang G., Malik V.S., Pan A., Kumar S., Holmes M.D., Spiegelman D., Lin X., Hu F.B. (2010). Substituting brown rice for white rice to lower diabetes risk: A focus-group study in Chinese adults. J. Am. Diet. Assoc.

[16] Jenkins D.J.A., Kendall C.W.C., Augustin L.S.A., Franceschi S., Hamidi M., Marchie A., Jenkins A.L., Axelsen M. (2002). Glycemic index: Overview of implications in health and disease. Am. J. Clin. Nutr.

[17] Miller A., Engel K.H. (2006). Content of γ-oryzanol and composition of steryl ferulates in brown rice (*Oryza sativa* L.) of European origin. J. Agric. Food Chem.

[18] Lu T.J., Chen H.N., Wang H.J. (2011). Chemical constituents, dietary fiber, and γ-oryzanol in six commercial varieties of brown rice from Taiwan. Cereal Chem.

[19] Jayadeep A., Malleshi N.G. (2011). Nutrients, composition of tocotrienols, tocopherols, and γ-oryzanol, and antioxidant activity in brown rice before and after biotransformation. CyTA J. Food.

[20] Roohinejad S., Mirhosseini H., Saari N., Mustafa S., Alias I., Shobirin M.H.A., Hamid A., Manap M.Y. (2009). Evaluation of GABA, crude protein and amino acid composition from different varieties of Malaysian’s brown rice. Aust. J. Crop Sci.

[21] Hu F.B., van Dam R.M., Liu S. (2001). Diet and risk of Type II diabetes: The role of types of fat and carbohydrate. Diabetologia.

[22] Ludwig D.S., Majzoub J.A., Al-Zahrani A., Dallal G.E., Blanco I., Roberts S.B. (1999). High glycemic index foods, overeating, and obesity. Pediatrics.

[23] Brand-Miller J., Hayne S., Petocz P., Colagiuri S. (2003). Low–glycemic index diets in the management of diabetes: A meta-analysis of randomized controlled trials. Diabetes Care.

[24] Bailey C.J., Turner R.C. (1996). Metformin. New Eng. J. Med.

[25] Edenberg H.J. (2007). The genetics of alcohol metabolism: Role of alcohol dehydrogenase and aldehyde. Alcohol. Res. Health.

[26] Ingelman-Sundberg M. (2005). Genetic polymorphisms of cytochrome P450 2D6 (CYP2D6): Clinical consequences, evolutionary aspects and functional diversity. Pharmacogenomics J.

[27] Pelkonen O., Turpeinen M., Hakkola J., Honkakoski P., Hukkanen J., Raunio H. (2008). Inhibition and induction of human cytochrome P450 enzymes: Current status. Arch. Toxicol.

[28] Mitschke D., Reichel A., Fricker G., Moenning U. (2008). Characterization of cytochrome P450 protein expression along the entire length of the intestine of male and female rats. Drug Metab. Dispos.

[29] Schmider J., Greenblatt D.J., von Moltke L.L., Karsov D., Shader R.I. (1997). Inhibition of CYP2C9 by selective serotonin reuptake inhibitors *in vitro*: Studies of phenytoin p-hydroxylation. Br. J. Clin. Pharmacol.

[30] Caraco Y., Muszkat M., Wood A.J. (2001). Phenytoin metabolic ratio: A putative marker of CYP2C9 activity *in vivo*. Pharmacogenetics.

[31] Lee C.R., Pieper J.A., Frye R.F., Hinderliter A.L., Blaisdell J.A., Goldstein J.A. (2003). Tolbutamide, flurbiprofen, and losartan as probes of CYP2C9 activity in humans. J. Clin. Pharmacol.

[32] Owens I.S., Ritter J.K., Yeatman M.T., Chen F. (1996). The novelUGT1 gene complex links bilirubin, xenobiotics, and therapeutic drug metabolism by encoding UDP-glucuronosyltransferase isozymes with a common carboxyl terminus. J. Pharmacokinet Biopharm.

[33] Levin B.E., Hogan S., Sullivan A.C. (1989). Initiation and perpetuation of obesity and obesity resistance in rats. Am. J. Physiol.

[34] Srinivasan K., Viswanad B., Asrat L., Kaul C.L., Ramarao P. (2005). Combination of high-fat diet-fed and low-dose streptozotocin-treated rat: A model for type 2 diabetes and pharmacological screening. Pharmacol. Res.

